# Oenological Characterization of Divona, a New Disease-Resistant Variety, and Preliminary Insights into the Influence of Maceration and Fermentation Temperature Across Three Vintages

**DOI:** 10.3390/foods15193358

**Published:** 2026-09-22

**Authors:** Marie Blackford, Ágnes Dienes-Nagy, Laurent Amiet, Marilyn Cléroux, Jean-Sébastien Reynard, Vivian Zufferey, Gilles Bourdin

**Affiliations:** 1 Agroscope, Enology Group, Route de Duillier 60, 1260 Nyon, Switzerland; 2 Changins, Viticulture and Oenology, HES-SO University of Applied Sciences and Arts Western Switzerland, Route de Duillier 52, 1260 Nyon, Switzerland; 3 Agroscope Viticulture Group, Avenue de Rochettaz 21, 1009 Pully, Switzerland

**Keywords:** fungus-resistant grape variety, white wine, Divona, pre-fermentation maceration, fermentation temperature, wine quality

## Abstract

Developing fungus-resistant grape varieties is essential to reduce phytosanitary inputs in viticulture. This study presents the first comprehensive oenological characterization of Divona, a new disease-resistant white grape variety developed by Agroscope. Musts and wines were produced over three contrasting vintages (2019, 2020, 2022) at the Pully experimental vineyard (Switzerland). Chemical analyses, sensory evaluations, and quantification of key aroma compounds (volatile thiols, monoterpenes) were conducted. A split-plot ANOVA and multiple factor analysis (MFA) separated vintage variability from winemaking effects (pre-fermentation maceration, fermentation temperature), which were treated exploratorily. The results indicate that vintage was the primary driver of variability. Lacking technical replicates within vintages, the study relies on biological replication across years to assess treatment robustness, interpreting winemaking effects as exploratory trends. Pre-fermentation maceration tended to enhance color intensity and certain aromatic precursors in the must. Under these specific conditions, the effects of fermentation temperature and maceration treatment appear modest relative to the dominant influence of vintage. Divona wines demonstrated high aromatic potential with a fruity expression. These findings establish a foundational oenological profile for Divona, validating its suitability for temperate climates and low-input viticulture, while offering preliminary technical guidelines to optimize sensory quality.

## 1. Introduction

Vines are particularly susceptible to fungal diseases. To ensure yield and grape quality, traditionally cultivated *Vitis vinifera* varieties require numerous phytosanitary treatments, the frequency of which depends on annual pathogen pressure and weather conditions. The deleterious effects of pesticide utilization on the environment and human health have prompted the European Union to establish an objective of reducing such use by transitioning from a treatment-based approach to one focused on disease prevention [1]. A key lever for this transition is the development of naturally resistant grape varieties, achieved through interspecific crosses between *Vitis vinifera* and wild species carrying resistance genes (e.g., *V. amurensis*, *V. riparia*) [2].

In Switzerland, Agroscope breeding programs have successfully developed such varieties, including the white grape variety Divona (approved in 2018), derived from a cross between Bronner and Gamaret [3]. Divona carries multiple resistance factors against downy mildew (RPV10 and RPV3), powdery mildew (REN3 and REN9), and black rot (RGB1), ripens early, and exhibits good sensory potential, often characterized by notes of exotic fruits, citrus, and flowers [4]. As one of the many new disease-resistant grape varieties currently available, Divona offers promising oenological potential for winegrowers. With the range of available grape varieties expanding, it is essential for winegrowers to have accurate information on the oenological characteristics of these new varieties. While several studies have explored the composition of certain white wines made from resistant grape varieties, the literature remains limited, particularly on the impact of certain oenological practices on wine composition and wine quality [5,6,7,8,9].

Among winemaking techniques, pre-fermentation maceration (PFM) and fermentation temperature are critical for modulating white wine profiles. PFM, involving skin contact at low temperatures (8–11 °C) for 6–24 h, generally enhances the extraction of aromatic precursors, potentially improving fruity and floral notes [10,11,12,13,14]. In addition to its impact on aromatic compounds, PFM induces significant changes in the phenolic composition of musts and wines. It often increases the content of total polyphenols, flavan-3-ols, and phenolic acids (vanillic, caffeic, and caftaric acids) and often improves antioxidant capacity [10,15,16,17,18]. However, it can also lead to increased bitterness, astringency, or the appearance of herbaceous characteristics, particularly in immature or damaged grapes [13,17,19]. PFM has also been associated with a decrease in total acidity, an increase in pH, and a decrease in the perception of acidity in the mouth [10,13]. The effects of PFM are highly dependent on temperature, duration, grape variety, vintage, and grape health [10,20]. With regard to resistant grape varieties, Skinkis et al. (2010) did not notice any difference between the aromatic profiles of ‘Traminette’, a US hybrid, wines that underwent a PFM at 12 °C for either 3 or 8 h [21]. Zhang et al. (2015) studied the influence of 6- and 24-h PFM on the Solaris grape variety in Denmark [22]. Their study showed an increase in amino nitrogen in the musts with 6- and 24-h PFM compared to direct pressing (DP). In the wine, there was an increase in alcohol (despite a non-significant increase in Brix in the must), with no differences in tartaric acid, pH and total acidity. There was an increase in total polyphenol content with PFM. In terms of aromatic compounds, wines with 24-h PFM had higher levels of certain esters such as ethyl decanoate (fruity), higher levels of C-6 alcohols, certain terpenes, and β-damascenone. Sensory analysis revealed that DP Solaris wine expressed more green vegetable and green berry notes, whereas pre-macerated wines expressed more fruity apricot notes.

Furthermore, temperature is a critical parameter during alcoholic fermentation, which typically occurs between 10 and 25 °C for white wines [12]. Low temperatures (15–18 °C) generally promote the production of volatile esters while reducing higher alcohols, resulting in a more fruity aromatic expression and a better balance between fruity and herbaceous notes [23,24,25,26,27]. However, excessively low temperatures risk altering yeast metabolism and causing sluggish fermentation [26,28]. Conversely, moderate temperatures (18–20 °C) can favor the formation of varietal thiols in sulfur-rich varieties [24,29,30]. Overall, temperature influences ethanol, organic acids (malic, succinic, acetic), glycerol, and phenolic compounds, with effects varying by variety, temperature range, and yeast strain [27,28,31,32,33,34]. Data on resistant varieties remains scarce; a study on Seyval Blanc showed that fermentation temperature affected acidity, ethyl acetate, total phenols, isobutanol, and ethyl lactate [35].

The use of resistant grape varieties is a concrete step towards more sustainable viticulture. However, for each of these new varieties, a blank page remains: how can they be vinified so that they fully express their potential and meet consumer expectations? The research pursued two hierarchical objectives: (1) to define the intrinsic chemical, aromatic, and sensory profile of Divona wines and (2) to provide preliminary insights into the modulating effects of pre-fermentation maceration and fermentation temperature on this profile. Conducted over three vintages, this study aims to identify the practices that best reveal the aromatic and sensory potential of this new resistant white grape variety.

## 2. Materials and Methods

### 2.1. Grape

Grapes used in this study were produced at Agroscope Pully (46°30′ N, 6°39′ E; 465 m asl), Vaud, Switzerland over three vintages (2019, 2020 and 2022). Due to yield losses caused by hail in 2021, the study could not be conducted using the 2021 vintage. The experimental site in Pully was on deep, clay (>15%) sandy soil without stones. The soil water holding capacity was estimated at 200 mm. Divona planted in 2010 and 2014 were grafted onto rootstock 3309C and were trained using a single Guyot training system (200 × 80 cm). During the vine growing season (April–October), the local average temperature was 16.6 °C in 2019, 16.9 °C in 2020 and 18.2 °C in 2022 and the total precipitation was 681 mm in 2019, 650 mm in 2020 and 539 mm in 2022 (Pully meteorological station, www.agrometeo.ch; accessed on 2 April 2026). The maximum recorded temperatures were 35.4 °C in 2019, 34.6 °C in 2020 and 35.9 °C in 2022. Grapes were harvested on 16 September 2019, 1 September 2020 and 24 August 2022. The 2020 and 2022 vintages were hot and relatively dry, while the 2019 vintage was an average vintage in terms of temperature and rainfall for the region.

### 2.2. Winemaking

Due to the scale of the trials, technical replicates within each vintage were not feasible. However, the repetition of the experiment over three distinct vintages (2019, 2020, 2022) serves as a biological replication to provide insight into the effects of treatment across varying climatic conditions.

For each vintage, the harvest from the plot was divided into two batches, supposedly homogeneous. For the first batch, corresponding to the direct pressing (DP) variant, the grapes were crushed, pressed in an Xpro 5 (Bucher Vaslin, Chalonnes-sur-Loire, France), cold settled (12 °C—36 h, 1 g/hL of Trenolin^®^ Kler P, Erbslöh, Geisenheim, Germany), and sulfited at a concentration of 50 mg/L. For the second batch, corresponding to the pre-fermentation maceration (PFM) variant , the grapes were crushed and placed in the Xpro 5, and the press was placed at 12 °C for 12 h. Then the same pressing procedure was applied, and the must was treated in the same way as the DP variant for settling and sulfiting. After the settling of the must, both juices were split into two tanks to perform the alcoholic fermentation (AF) either at 15 °C or at 20 °C. Fermentation temperature was controlled using a cooling coil. Approximately 90 L were vinified per experimental modality. AF was started with 20 g/hL yeast addition (CY3079, Lallemand Inc., Montréal, QC, Canada), without nitrogen correction, and was monitored daily by density measurement. At the end of AF, the wines were fined (15 mL/hL KlarSol Super, Erbslöh; 60 mL/hL IsingClair Hausenpaste, Erbslöh), filtered, stabilized with 50 mg/L SO_2_, and stored for one month at 0 °C for tartaric stabilization. Three months later, they were filtered with a 0.65 µm filter and bottled. Bottles were stored under controlled conditions (10–12 °C, in a dark room) until the analysis.

### 2.3. Chemical Analysis

#### 2.3.1. Must and Wine General Composition

Must samples were collected from the tank before fermentation, and standard composition parameters (sugar content (Brix), pH, total acidity (g tartaric acid/L), tartaric acid (g/L), and malic acid (g/L)) were determined by infrared spectroscopy technology (FTIR WineScanTM, FOSS, Hillerød, Denmark). The decrease in sugar content in Oeschle (°Oe) during AF was measured using a densitometer DMA35 (Anton Paar, Graz, Austria). The ammonium nitrogen (NH_3_) and amino acid nitrogen (N_AA_) were determined on an A25 spectrophotometric autoanalyzer (Bio System, Barcelona, Spain), using commercial methods: an enzymatic one for ammonium NH_3_ (Methods of Biochemical Analysis and Food Analysis, Boehringer Mannheim GmbH, Mannheim, Germany, 1997) and a spectrophotometric one with a dedicated kit for free primary amino acids (“Primary Amino Nitrogen” from Bio Systems, Barcelona, Spain), which uses δ-phtalaldehyde/N-acetyl-cysteine as the reagent (also called the NOPA method). YAN was calculated as the sum of nitrogen (mg N/L) in the form of NH_3_ and N_AA_.

Three months after bottling, the classical wine parameters were analyzed with FTIR WineScanTM (FOSS, Hillerød, Denmark) (ethanol (%vol), dry extract (g/L), pH, total acidity (g tartaric acid/L), glycerol (g/L), tartaric acid (g/L) and malic acid (g/L)).

The color measurements were performed on an Agilent Cary 60 spectrophotometer using the CIELAB uniform color space (Agilent Technologies, Santa Clara, CA, USA).

#### 2.3.2. Volatile Thiols and Their Precursors

a. 3-mercaptohexanol precursors

Precursors of 3-mercaptohexanol (3MH), namely 3-mercaptohexanyl-cystein (3MH-Cys) and 3-mercaptohexanyl-glutathion (3MH-Glu) were quantified in the must. Must samples were centrifuged at 9000 rpm for 5 min to remove particulate matter. The resulting supernatant was transferred into HPLC vials for analysis. S-benzyl cysteine (1 ng/L in acetonitrile:water, 1:200) was used as the internal standard (IST). Chromatographic separation was performed using an Agilent Infinity 1290 UPLC system (Agilent Technologies, Santa Clara, CA, USA), which included a quaternary pump (Pump 1), an isocratic pump (Pump 2), an injector, and a column compartment equipped with a 10-position column selection valve. The UPLC system was coupled to an Agilent 6460 Triple Quadrupole LC-MS (Agilent Technologies) operating in electrospray ionization positive mode (ESI+), utilizing Agilent Jet Stream technology and controlled by MassHunter Software(v10). Two columns were used in the analysis: a Pinnacle DB AQ C18 column (50 × 2.1 mm, 1.9 μm; Restek, Bellefonte, PA, USA) installed in port 2 (pre-column) and a Poroshell 120 Bonus RP column (150 × 2.1 mm, 2.7 μm; Agilent Technologies) installed in port 10 (Figure A1). The mobile phase consisted of: (A) water with 0.1% formic acid and (B) acetonitrile with 0.1% formic acid. The flow rate was set at 0.3 mL/min. A sample volume of 5 μL (4 μL of must and 1 μL of IST) was injected. The injection was first directed to the pre-column (Port 2) for 4 min to eliminate sugars, after which the valve was switched to Port 10 for chromatographic separation. The elution gradient was as follows: from 0 to 4 min, 2% of B; from 4 to 10 min, a linear gradient to arrive at 100% of B; from 10 to 12 min, 100% of B. The column was equilibrated for 4 min with 2% of B. Detection was performed by multiple reaction monitoring (MRM), monitoring the following mass transitions: 222 *m*/*z* → 83 *m*/*z* for 3MH-Cys, 408 *m*/*z* → 162 *m*/*z* for 3MH-Glu, and 212 *m*/*z* → 91 *m*/*z* for S-benzyl cysteine (IST). Electrospray positive ionization mode (ESI+) was applied with the following parameters in the source: gas temperature at 300 °C, gas flow at 5 L/min, nebulizer at 45 psi, sheath gas heater at 250 °C, sheath gas flow at 11 L/min, capillary voltage at 3500 V, and nozzle voltage at 500 V. The fragmentor voltage and collision energy were optimized with standards separately and were found to be 73 V and 4 V for 3MH-Cys, 81 V and 20 V for 3MH-Glu, and 63 V and 20 V for S-benzyl cysteine (IST), respectively. Quantification was performed using a calibration curve obtained with standard solutions of 3MH-Cys, 3MH-Glu, and S-benzyl cysteine in the range of 5–200 μg/L. The LOD of the method is 1 μg/L, the LOQ is 5 μg/L, CV% < 5%, and recovery is 82–105%.

b. 3-mercaptohexanol and 4-mercapto-4-methylpentan-2-one (4-MMP)

The method reported by Capone et al. (2015) was adapted for the analysis of 3MH. The analysis was performed on an Infinity 1290 UPLC system (Agilent Technologies, Santa Clara, CA, USA) connected to an Agilent 6460-C Triple Quadrupole LC-MS with an electrospray using Agilent Jet Stream technology and operating under MassHunter Software (Agilent Technologies, Santa Clara, CA, USA) [36]. Chromatographic separation was performed on a Poroshell 120 SB-C18 column (150 × 4.6 mm, 2.7 μm; Agilent N°683975-902) operated at 40 °C. We injected 100 µL of wine sample directly and derivatized it in situ on the column using a 0.1 mM solution of 4,4-dithiopyridine (DTDP) in ammonium formate buffer (100 mM, pH 4.2), which was used as mobile phase A with a flow rate of 0.5 mL/min. The mobile phase B was acetonitrile, and the elution gradient was as follows: from 0 to 12 min, 5% of B; from 12 to 34 min, a linear gradient to reach 100% of B; 100% of B from 34 to 36 min; and from 36 to 38 min, a linear gradient to come back to 5% of B. The column was equilibrated for 12 min with 5% of B between injections. Detection was performed using MRM. ESI+ was applied with the following parameters in the source: gas temperature at 300 °C, gas flow at 5 L/min, nebulizer at 45 psi, sheath gas heater at 250 °C, sheath gas flow at 11 L/min, capillary voltage at 3500 V, and nozzle voltage at 500 V. The fragmentor voltage and collision energy were optimized with standards for 3MH and 4-MMP and were found to be respectively 100 V and 110 V for the fragmentor and 15 V for both compounds for the collision energy. The flow was directed after the column to the waste in the first 28 min of the run and to MS between 28 and 32 min. Quantification was performed using the following transitions: 244 *m*/*z* → 144 *m*/*z* for 3MH and 242 *m*/*z* → 144 *m*/*z* for 4-MMP. Concentration was determined using external calibration with standards in the range of 50–2000 ng/L for 3MH and 5–200 ng/L for 4-MMP. The LOD is 10 ng/L and 1 ng/L, the LOQ is 25 ng/L and 5 ng/L, CV% < 7% and 10%, and recovery is between 92 and 100% respectively for 3MH and 4-MMP.

#### 2.3.3. Terpene Analysis

Free and glycosylated monoterpenes were measured following the method of Lan et al. (2016), as previously published [37].

### 2.4. Sensory Analysis

A sensory analysis was conducted three months after bottling by a panel of 12 experienced tasters who had undergone annual training to standardize the evaluation of the 14 sensory attributes (including wine appearance, bouquet and palate). Wines were evaluated twice on the same day, across two separate tasting series; panelists rated the intensity of the different attributes on an unstructured line scale from 1 (no perception) to 7 (very intense perception). For each wine, a 50 mL sample was served at 17 ± 1 °C in transparent INAO glasses, anonymized with three-digit codes, and presented in randomized orders for each panelist. The results were collected with the FIZZ software (version 2.61, Biosystèmes, Couternon, France).

### 2.5. Statistical Analysis

All statistical analyses were performed using R software (v4.6.1). Fermentation kinetics were analyzed from 162 repeated observations nested within 12 fermentation tanks (TankID = maceration × temperature × vintage). Because sugar decrease during fermentation typically follows a sigmoidal pattern, a non-linear mixed-effects model (nlme package) was fitted using a four-parameter logistic function,

(1) $$Sugar(t)=A+\frac{B-A}{1+exp(\frac{x_{mid}-t}{\tau })},$$

where A and B are the upper and lower asymptotes, respectively, x_mid_ is the inflection day, τ is the transition rate, and t is time (days after yeasting). Asymptotes (A, B) were held common across tanks; x_mid_ and τ varied by temperature (15 °C vs. 20 °C), maceration (DP vs. PFM), and their interaction, with a random intercept on x_mid_ and τ grouped by TankID. Treatment effects on curve shape were tested via likelihood-ratio tests (LRT) comparing the full model to reduced models excluding each fixed effect. As a complementary time-resolved visualization, temperature differences were also tested on each day using linear models (Sugar~Temperature + Maceration) with emmeans comparisons, and *p*-values were corrected using the Benjamini–Hochberg (BH) procedure across days. The results are shown as mean ± SE with the model’s fitted curves overlaid; asterisks show day-specific comparisons (* *p* < 0.05, ** *p* < 0.01, *** *p* < 0.001), while global LRT results are reported in the figure caption.

For the must dataset, fermentation temperature could not be considered as a factor, since temperature control was only applied after the must had been split into sub-lots for alcoholic fermentation; must parameters were therefore analyzed with respect to maceration treatment (DP vs. PFM) only, with one tank per treatment and per vintage (3 vintages, no technical replicates). Given this minimal sample size, a formal analysis of variance was not considered appropriate. Instead, the PFM vs. DP difference was calculated for each parameter within each vintage, and the consistency of this difference across the three vintages was used as the primary descriptive criterion to evaluate the direction and consistency of the maceration effect.

For the wine dataset, each parameter was analyzed independently using a split-plot analysis of variance (ANOVA), with maceration treatment (DP vs. PFM) as the whole-plot factor, fermentation temperature (15 °C vs. 20 °C) as the sub-plot factor, and vintage (2019, 2020, 2022) as the blocking factor defining the whole-plot error stratum. The model formula was specified as

(2) $$Y_{ijk}=\mu +V_{j}+M_{j(i)}+T_{k}+{\left(M\times T\right)}_{jk}+\epsilon_{ijk},$$

with Y as the parameter, V as the vintage, M as the maceration, and T as the AF temperature. The model was fitted using the aov() function:

aov(Parameter~Maceration * AF temperature + Error(Vintage/Maceration)).
(3)

This error structure partitions the total variability into three strata: (i) vintage (whole-plot block), (ii) maceration nested within vintage (whole-plot error, used to test the maceration effect), and (iii) the residual (sub-plot) stratum, used to test the temperature effect and the maceration × temperature interaction. In the absence of technical replicates within each vintage × treatment combination, the residual variance of the maceration stratum (nested within vintage) was used as the error term to test the maceration effect. Given this constraint, the winemaking technique effects (maceration, temperature, and their interaction) were interpreted as exploratory trends rather than confirmatory results.

For each parameter, Type I sums of squares were used, consistent with the sequential nature of the split-plot error decomposition. *p*-values for the maceration, temperature, and maceration × temperature effects were extracted from the corresponding ANOVA strata and corrected for multiple testing across the parameters using the Benjamini–Hochberg (BH) procedure, applied separately to each of the three effects. Residuals from the sub-plot (Within) stratum were visually inspected for each model using quantile–quantile plots and residuals-versus-fitted-values plots to assess normality and homoscedasticity assumptions.

Sensory scores were analyzed using a linear mixed-effects model (LMM) to account for panelist variability and the repeated-measures nature of the data. The model included vintage, maceration, AF temperature, their interaction (maceration × AF temperature), and tasting series (Serie) as fixed effects. Panelist and TankID were included as random intercepts to account for individual judge variability and batch-to-batch variability among replicate vinifications. The model formula was

(4) $$Y_{ijklmn}=\mu +V_{i}+M_{j}+T_{k}+{\left(M\times T\right)}_{jk}+S_{l}+u_{m}+w_{n}+\epsilon_{ijklmn},$$

with Y as the sensory score, V as the vintage, M as the maceration, T as the AF temperature, S as the tasting series (Serie), u_m_ as the random intercept for Panelist, and w_n_ as the random intercept for TankID. This model was implemented in R using the lmer() function (package lmerTest):

lmer(Sensory score~Vintage + Maceration * AF temperature + Serie (1|Panelist) + (1|TankID)).
(5)

When the random effect variance associated with Tank ID was estimated at or near zero, leading to a singular model fit, the model was refitted without this random term, retaining only Panelist as a random effect. *p*-values for the fixed effects were obtained using Satterthwaite’s approximation for degrees of freedom (as implemented in lmerTest) and corrected for multiple testing across sensory attributes using the Benjamini–Hochberg (BH) procedure, applied separately for each effect (vintage, maceration, AF temperature, interaction).

Multiple factor analysis (MFA) was performed using the FactoMineR (v2.16) and factoextra packages( v2.2.0) to integrate physicochemical, aromatic, and sensory variable groups and to visualize the overall structure of the data and the relationships between individuals and variables [38]. Climate conditions (mean temperature, rainfall, and max temperature for each vintage) were projected as supplementary variables. This approach allowed us to interpret the factorial structure without influencing the construction of the axes, thereby assessing how climatic patterns correlate with the variability defined by the groups of variables.

### 2.6. IA Use

Euria (v1.2.2, Infomaniak AI Assistant, Geneva, Switzerland), a GenAI, was used for the purposes of drafting, summarizing, and revising the text and the R code for the statistical analysis.

## 3. Results

### 3.1. Must Analysis

Divona musts exhibited sugar concentrations ranging from 22.5 to 24.2 °Brix (Table 1). Total acidity varied between 4.3 and 5.7 g/L (expressed as tartaric acid), with pH values spanning from 3.32 to 3.65. Malic acid content was notably low across all samples, remaining below 1.2 g/L. Regarding assimilable nitrogen, the Divona musts contained between 251 and 302 mg N/L, predominantly in the form of amino nitrogen (N_AA_). Finally, analysis revealed the presence of volatile thiol precursors 3MH-Glu (77%) and 3MH-Cys (23%).

The influence of the winemaking techniques (DP vs. PFM) on must composition was also evaluated. Given the limited number of independent replicates (n = 3 vintages per modality), these results are presented as consistent trends across contrasted vintages rather than as formally significant statistical effects. PFM was consistently associated with lower sugar content, total acidity, and tartaric acid and higher pH, compared to DP, across all three vintages studied. Nitrogen in the NH_3_ form was also consistently higher under PFM. The most pronounced and consistent result was observed for thiol aroma precursors, with 3MH-Cys and 3MH-Glu markedly increasing under PFM in all three vintages (mean increase of 47.7 mg/L and 60.6 mg/L, respectively), indicating a tendency for increased varietal thiol precursor content associated with this maceration technique. Interestingly, the large standard deviations for the two thiol aroma precursors highlight a strong vintage influence on these compounds. In contrast, malic acid, amino acid nitrogen (N_AA_) and yeast assimilable nitrogen (YAN) showed a less consistent response, with one vintage diverging from the majority trend, and should therefore be interpreted with additional caution.

### 3.2. Alcoholic Fermentation Kinetics

The kinetics of sugar consumption, in Oechsle, were modeled using a non-linear mixed model (NLME) with a logistic sigmoid function (four parameters), which converged for all 12 tanks. Curves are shown in Figure 1. Density values below zero were observed as a natural consequence of alcohol accumulation, since ethanol is less dense than water. The estimated fixed parameters indicate an average initial sugar content (A) of 112.5 ± 1.5 °Oe and a final residue (B) of −8.6 ± 0.5 °Oe (Table A1). All wines fermented completely.

LRT revealed a highly significant effect of temperature on the overall shape of the curve (χ^2^ = 22.74, *p* < 0.001), in contrast to maceration (χ^2^ = 4.09, *p* = 0.39). No significant interaction was detected (*p* > 0.21). Fermentation at 20 °C significantly accelerated the rate of consumption (τ parameter: +0.36, *p* < 0.001) and brought the inflection point (x_mid_) forward by 0.57 days compared with 15 °C (*p* < 0.001; Table A1). Consequently, samples fermented at 20 °C demonstrated a more rapid fermentation rate, reaching sugar stability by day 9, whereas those fermented at 15 °C exhibited a flatter curve, fermented more slowly, and completed fermentation around day 13. Pairwise comparisons confirmed a significant divergence between the temperatures from day 3 to day 10 (*p* < 0.05), with the most pronounced differences observed between days 6 and 8 (*p* < 0.002; Table A2). Notably, the PFM-15 °C modality showed the greatest variability between vintages.

### 3.3. Wine Composition

The wines were analyzed 3 months after bottling, and Table 2 presents the average values for all winemaking modalities and vintages, providing a general overview of the composition of Divona wines. Divona wines exhibited alcohol content of around 14% by volume, with total acidity values below 5 g/L and pH values ranging from 3.38 to 3.67. The CIELab coordinates indicated a wine of high lightness (L* = 99.1 ± 0.9). The color intensity was low (C* = 7.3 ± 2.1). The hue (H* = 100.2 ± 0.7) positioned the color in the yellow-green quadrant, indicative of a young white wine with greenish reflections. This was supported by the positive b* value (7.2 ± 2.0), denoting a yellow tonality, while the slightly negative a* value (−1.3 ± 0.3) suggested a minor green component. The relatively high standard deviation observed for the a* coordinate and C* highlighted a notable variability in color intensity and red nuance across vintages and modalities. Regarding the aromatic composition, the wines presented 3MH concentrations ranging from 104.4 to 613.0 ng/L (mean: 246.9 ± 223.3 ng/L), while 4MMP was not detected in any sample. Several terpenes were also identified, with mean concentrations of cis-linalool oxide (8.6 ± 6.4 µg/L), trans-linalool oxide (11.1 ± 8.5 µg/L), linalool (35.8 ± 25.8 µg/L), α-terpineol (17.9 ± 6.6 µg/L), β-citronellol (17.5 ± 2.9 µg/L), nerol (24.1 ± 20.1 µg/L), and geraniol (81.1 ± 48.9 µg/L). The substantial standard deviations associated with these aromatic compounds, particularly for 3MH, linalool, nerol, and geraniol, further underscore the significant variability driven by vintage conditions.

The investigation of winemaking techniques using a split-plot ANOVA across three vintages revealed no statistically significant effect of maceration treatment (DP vs. PFM) on the measured chemical or aroma-related parameters after correction for multiple testing (Table 2). Fermentation temperature emerged as the primary driver of variation, exerting a statistically significant effect on alcohol content and wine pH across the independent technological replicates (two tanks per vintage). Wines fermented at 20 °C exhibited marginally higher alcohol concentrations than those at 15 °C (+0.25 to +0.30% vol), alongside slight increases in pH (+0.03 to +0.05 units). Regarding other compounds, no significant influence of fermentation temperature was detected.

### 3.4. Wine Sensory Analysis

Sensory evaluation, carried out on a seven-point scale, revealed a profile dominated by structural and fruity attributes (Table 3). Acidity had the highest intensity (4.6 ± 0.5), closely followed by color intensity (4.6 ± 0.5) and bouquet quality (4.5 ± 0.5). Descriptors such as equilibrium (4.4 ± 0.5), overall appreciation (4.4 ± 0.5) and fruity character (4.4 ± 0.6) showed high average scores. The volume on the palate was also well defined (4.1 ± 0.4).

Conversely, secondary and tertiary notes have the lowest intensities. The buttery/milky (1.2 ± 0.4) and empyreumatic (1.2 ± 0.5) characteristics are virtually absent from the profile. Similarly, the mineral (1.5 ± 0.6) and stress (1.5 ± 0.7) notes remain subtle. Vegetal attributes (1.9 ± 0.8) and bitterness (2.4 ± 0.8) are at the lower end of the scale, whilst the floral character (3.0 ± 0.9) occupies an intermediate position with slightly greater variability.

Sensory evaluation of the wines by the expert panel revealed no significant effect of maceration, AF temperature, or their interaction on any of the 14 attributes assessed after BH correction for multiple testing (all adjusted *p*-values > 0.6 for maceration; all adjusted *p*-values = 0.973 for AF temperature; all adjusted *p*-values > 0.1 for the maceration × AF temperature interaction). Bitterness showed the lowest interaction *p*-value among all attributes (adjusted *p*-value = 0.157), but this did not approach statistical significance. These results indicate that, within the range of vintages and winemaking conditions tested, the sensory profile of Divona wines as perceived by the expert panel was not measurably affected by maceration technique or fermentation temperature, whether individually or in combination.

### 3.5. Multiple Factor Analysis

To explore simultaneously the variations in the different data sets, an MFA was performed to highlight the relationships between individuals and groups of variables. Climate conditions were also projected as supplementary information (mean temperature, rainfall and max temperature for each vintage). The two first dimensions of the MFA explain 41.9% and 28.6% of the total variance, respectively, and were selected for the interpretation of the results. The representation of individuals on the factorial plane made it possible to visualize the distribution and the 95% confidence ellipses that highlight the separation of groups, suggesting potential influence of maceration, AF temperature and vintage (Figure 2). The confidence ellipses associated with oenological techniques, maceration and AF temperature largely overlap, suggesting little differentiation between samples according to these factors. On the other hand, the ellipses corresponding to vintages are clearly separated in terms of factors, indicating that vintage is a major discriminating factor within the data set.

According to the graph of the variables, for the first dimension (41.9% of the variance), positive values are associated with increased color intensity (C*, b*, color intensity), more pronounced bitterness and more pronounced mineral and empyreumatic notes in the bouquet (Figure 3). It is also in this direction that mean temperatures, a supplementary variable, are projected. Conversely, negative values for Dim 1 correspond to higher levels of total acidity, nerol concentration, floral notes in the bouquet, higher perceived acidity, and a lighter color (L*, a*). Annual rainfall, as a supplementary variable, is also projected on this side of Dim 1.

The second dimension (28.6% of the variance) mainly distinguishes between aromatic compounds and specific must and wine compounds. Positive values are strongly correlated with terpenes (linalool, geraniol, α-terpineol), 3MH, must nitrogen richness (YAN), tartaric acid and alcohol content, reflecting a more pronounced varietal aromatic expression and more advanced aromatic maturation. Conversely, negative values for Dim 2 are associated with higher levels of malic acid, the presence of oxidized linalool derivatives and higher maximum daily temperatures (supplementary variable).

## 4. Discussion

Divona is an early-ripening grape variety, judging by the harvest dates (16 September 2019, 1 September 2020, and 24 August 2022), especially in hot years such as 2020 and 2022. This confirms what has already been mentioned by Mackie-Haas et al. [4]. At harvest time, sugar levels range from 22.5 to 24.2 °Brix. These values are generally in the high range for white wines but within the range of data published on resistant grape varieties, which have sugar contents ranging from 14.8 to 30.5 °Brix [8]. Similar values have been found for other resistant white grape varieties grown in Spain [5], with values in the musts at harvest from 23.5 to 24.7 °Brix depending on the vintage for the Sauvignon Kretos variety, from 26 to 27 °Brix for Souvignier gris, and from 28.4 to 29.5 °Brix for Muscaris, also considered an early variety. In comparison, studies on Sauvignon Blanc generally show must sugar contents between 19 and 22 °Brix [24,30]. Nevertheless, these comparisons should be considered indicative, given that the production climate has a major impact on this parameter. In terms of acidity and pH, Divona is a grape variety with relatively low acidity. Although maturity monitoring has not been carried out on Divona, it can be assumed that early ripening would also lead to a decrease in acidity, particularly malic acid, but this remains to be confirmed. The pH of Divona wines measured was between 3.4 and 3.7, with total acidities below 5 g of tartaric acid/L. Although these values are similar to those found for Sauvignon Kretos, Souvignier gris, Muscaris, and Sauvignon Blanc wines in the study by Casanova-Gascon et al. [5], a high alcohol content combined with low acidity can lead to sensory imbalance. Indeed, high ethanol content has been shown to be responsible for increased heat in the mouth in Riesling wines [39], as well as for the perception of bitterness [40]. These two aspects suggest that Divona is a grape variety that seems more suited to cooler terroirs, which will slow its ripening and limit sugar load and acidity loss. In terms of assimilable nitrogen content, the values observed in Divona musts are well above 140 mg N/L, with the threshold generally considered to indicate nitrogen deficiency [41]. The problems identified by Duley et al. [8], concerning excessive nitrogen in resistant grape varieties, do not apply to Divona. Although the YAN values are similar for Solaris in the study by Zhang et al. [22], the distribution between ammonium and amino acids differs: while in Divona, amino nitrogen (N_AA_) represents about 96% of the assimilable nitrogen, in Solaris it represents only about 70%. The influence of the nitrogen source during fermentation, particularly amino acids, can have an impact on the volatile composition of wines [42,43].

In the study by Nicolle et al. [44], and in that by de Almeida et al. [6], analyzing thiol precursors in different resistant white grape varieties, the amount of 3MH-Glu was always higher than the amount of 3MH-Cys, which is also the case for Divona. 3MH-Glu is also the main precursor for traditional *Vitis vinifera* grape varieties [45]. In any case, the 3MH values found in Divona wines are above the perception threshold of 50 and 60 ng/L in hydroalcoholic model solution [46] and similar to those found in Sauvignon Blanc wines. It is interesting to note that Divona wines did not contain 4-mercapto-4-methylpentan-2-one (4MMP), unlike Sauvignon Blanc [47]. With regard to terpene analysis in wines, the linalool values of Divona wines were above the perception threshold of 25.2 µg/L [48]. The same is true for geraniol, which has a perception threshold of 30 µg/L. The values for α-terpineol and β-citronellol are below the perception thresholds of 250 µg/L and 100 µg/L, respectively [48]. These terpene content values are similar to those found in other white grape varieties such as Chardonnay, Sauvignon Blanc, and certain Muscat varieties, but lower than Riesling or Gewurztraminer [48]. The terpene composition of musts and wines is highly dependent on abiotic factors; the vintage effect plays a decisive role. On Dimension 2, the MFA reveals opposing trends: maximum daily temperature aligns with cis- and trans-linalool oxides but opposes linalool, geraniol, and α-terpineol. This negative association for volatile terpenes is consistent with the literature reporting that intense heat (>35 °C) during ripening can degrade terpene content [49]. Nevertheless, given the exploratory nature of the MFA and the limited number of vintages, these visual patterns should be interpreted as hypothetical links pending further confirmation.

PFM induced a decrease in total acidity and tartaric acid and an increase in the pH of the musts. The decrease in tartaric acid with PFM has already been observed in the literature and is generally associated with greater potassium extraction from grape skin [13]. It would be interesting to measure the potassium content to confirm this hypothesis. Changes in acidity were not observed in wines, and no significant effect was detected at the sensory level in this study. However, this absence of significance should not be interpreted as a lack of effect, but rather attributed to the limited statistical power of the experiment. Specifically, the lack of technical replicates per vintage for the maceration treatment reduces the ability to detect subtle differences, meaning that potential impacts on acidity analysis and perception cannot be ruled out. Furthermore, various stages of winemaking, such as tartaric stabilization, can influence the overall acid balance of wines. Consequently, the perception of acidity in wines is not solely explained by laboratory measurements and depends on several factors [50].

In terms of volatile thiols, PFM successfully increased the concentration of nonvolatile precursors (3MH-Cys and 3MH-Glu) in the musts, indicating its efficacy in the extraction stage. However, this increase did not translate into higher 3MH concentrations in finished wines, indicating a lack of direct correlation between precursor availability and final aromatic expression. This outcome underscores the critical role of the yeast-mediated conversion stage during alcoholic fermentation. The literature indicates that the average level of conversion of precursors into their volatile thiols is relatively low (4.2% for 3MH-Cys) and highly dependent on fermentation conditions, particularly the yeast strain and oxygen levels [45]. Furthermore, the large standard deviation observed in wine 3MH values suggests that retention or loss mechanisms post-fermentation, likely driven by vintage-specific variability, also play a decisive role. Consequently, the primary practical conclusion of this study is that increasing sulfur-containing precursor concentrations via PFM is not sufficient to guarantee elevated volatile thiol levels in Divona wines across all vintages. Rather than focusing solely on precursor extraction, future strategies should investigate optimizing the conversion phase, potentially through the use of specific thiol-revealing yeast strains, to achieve more stable and pronounced results across vintages.

The effect of lower AF temperature (15 °C) induced lower alcohol and pH values. While the presence of these replicates provides sufficient statistical power to detect these differences as significant, their practical oenological relevance warrants careful consideration: such magnitudes often fall within the range of analytical variability or may remain below the sensory perception threshold for most consumers. Thus, while temperature is confirmed as the dominant factor relative to maceration in this design, its impact on the final wine profile under these specific conditions appears modest.

Sensory evaluation revealed a wine profile dominated by fruity aromas, a description consistent with the existing literature for this cultivar [3,4]. No significant effects stemming from the winemaking techniques (maceration or AF temperature) were detected on the sensory attributes in this study. Notably, the standard deviations were particularly large for floral notes, underscoring that vintage-specific variability exerts a stronger influence on the aromatic profile than the oenological treatments applied. However, consistent with our interpretation of the chemical results, this lack of statistical significance in the sensory analysis should not be viewed as a definitive absence of effect. It primarily reflects the limited statistical power due to the lack of technical replicates. Therefore, while vintage effects dominate, subtle sensory impacts of PFM and temperature modulation cannot be entirely excluded.

## 5. Conclusions

This study has generated new information, particularly regarding the composition of Divona wines, a new grape variety resistant to fungal diseases and with good quality potential. This relatively early-ripening variety appears to be suited to cooler terroirs, which will limit or slow its ripening and ensure a certain level of acidity and correct sugar levels. Containing 3MH thiols and various terpenes, it has a pleasant, fruity and floral bouquet. The strong impact of the vintage shows that monitoring the ripening of the grapes and harvesting them while retaining a certain level of acidity are essential. PFM appeared to be efficient in increasing nonvolatile thiol precursors (3MH-Glu and 3MH-Cys); however, no direct correlation was found between precursor availability and their conversion into 3MH. Additionally, Divona wines did not contain detectable levels of 4MMP thiols. While vintage variability had the strongest impact on both wine composition and sensory profile, the winemaking techniques, as tested in this study, showed no significant effect on either parameter.

## Figures and Tables

**Figure 1:**
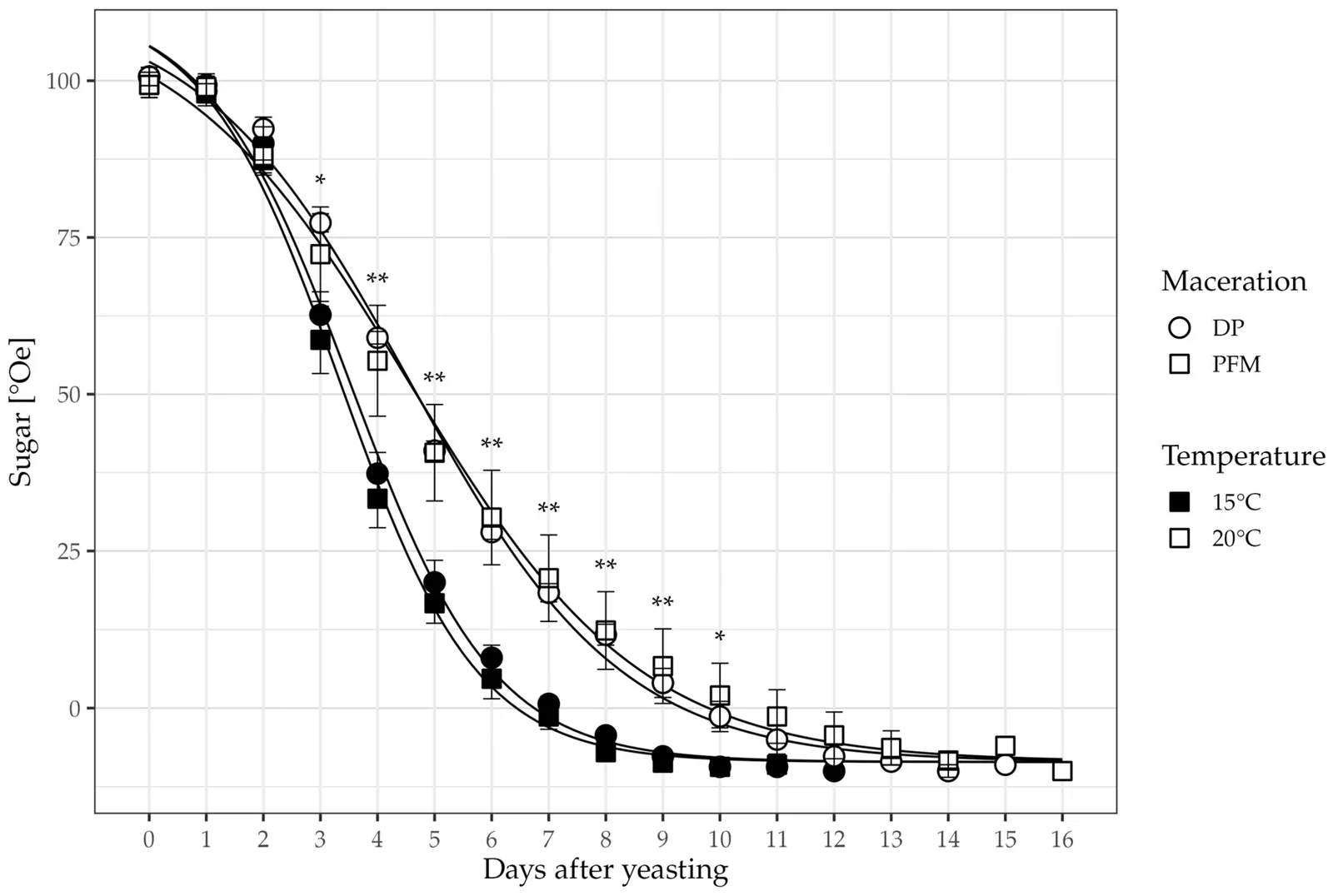
Comparison of fermentation kinetics fitted with non-linear mixed model according to the different winemaking modalities; DP = direct pressing; PFM = pre-fermentation maceration; 15 °C/20 °C = alcoholic fermentation temperature (mean of three vintages, 2019, 2020 and 2022); Error bars represent the standard deviation between vintages; overall curve-shape LRT: AF temperature *p* = 0.00014, maceration *p* = 0.39; asterisks indicate BH-adjusted pairwise temperature comparisons for each day (* *p* < 0.05, ** *p* < 0.01).

**Figure 2:**
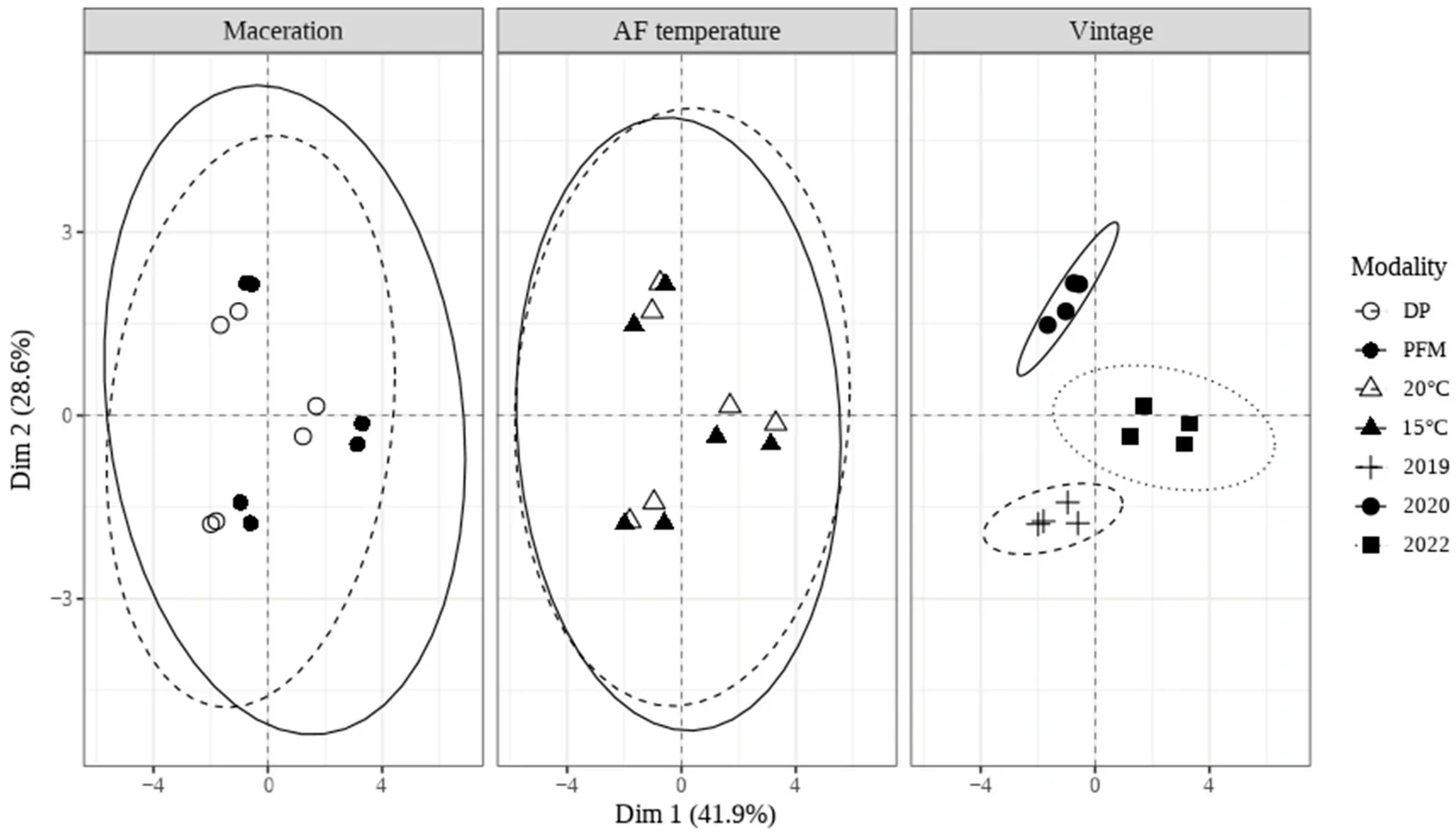
MFA—individual map faceted by the supplementary variables maceration, AF temperature and vintage (with 95% confidence ellipses).

**Figure 3:**
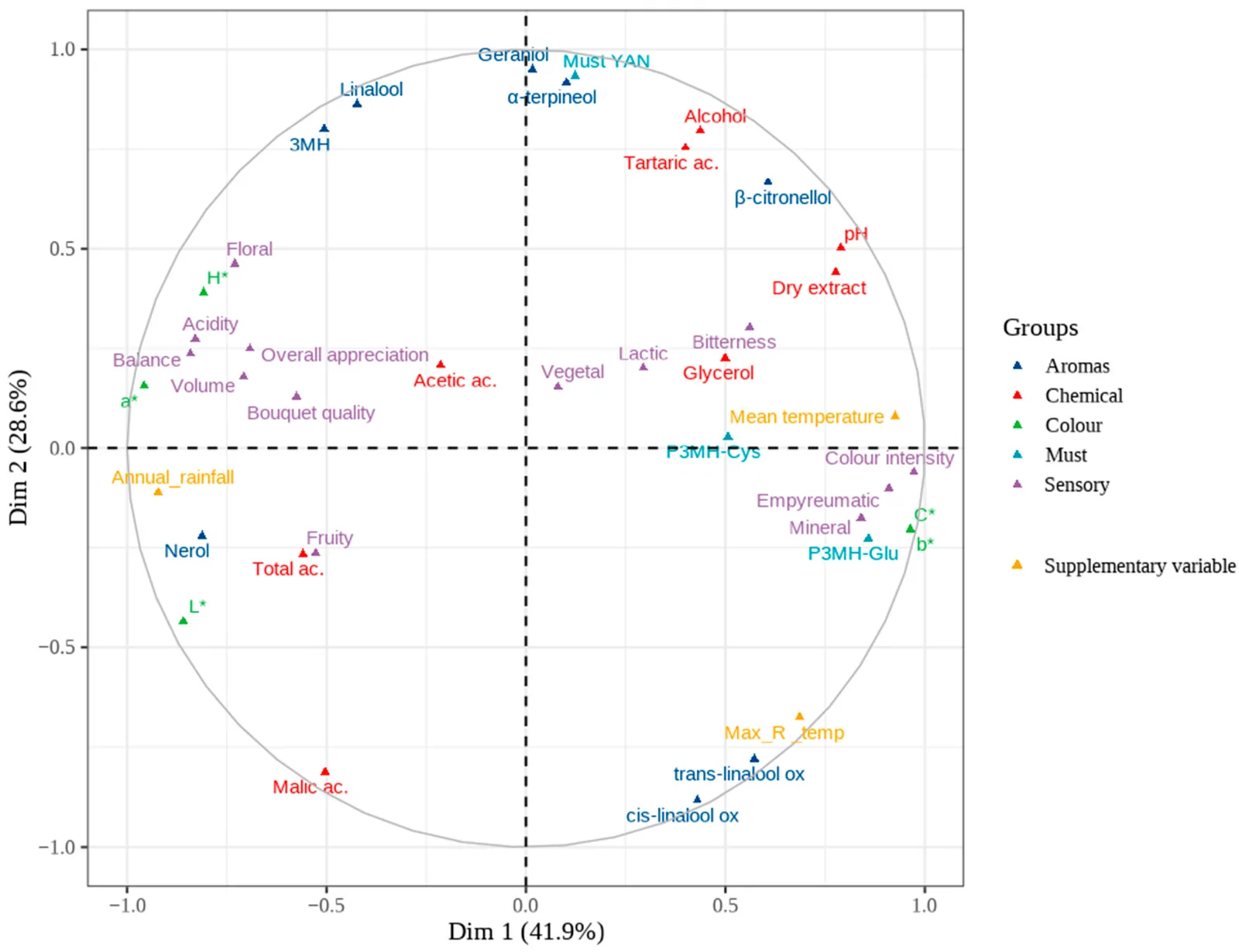
MFA—variable representation.

**Table 1:** Effect of PFM compared to DP on the chemical composition of Divona musts comparison for three vintages (2019, 2020 and 2022).

Parameter	DP	PFM	Δ (PFM-DP)	Positive/Negative	Robustness (n = 3)
	Mean ± SD	Mean ± SD	Mean ± SD		
General composition					
Sugar content (°Brix)	23.5 ± 0.8	23.4 ± 0.9	−0.1 ± 0.1	0/3	Consistent (3/3)—decrease
Total acidity ^1^ (g/L)	5.4 ± 0.4	4.5 ± 0.2	−0.9 ± 0.2	0/3	Consistent (3/3)—decrease
pH	3.44 ± 0.11	3.59 ± 0.10	0.16 ± 0.05	3/0	Consistent (3/3)—increase
Tartaric acid (g/L)	7.1 ± 0.4	6.1 ± 0.2	−1.0 ± 0.2	0/3	Consistent (3/3)—decrease
Malic acid (g/L)	1.0 ± 0.2	0.9 ± 0.1	−0.1 ± 0.2	1/2	Not fully consistent (2/3)
Nitrogen compounds					
NH_3_ (mg/L)	11.7 ± 2.1	12.1 ± 2.3	0.3 ± 0.2	3/0	Consistent (3/3)—increase
N_AA_ (mg/L)	260.1 ± 14.3	266.3 ± 21.7	6.1 ± 15.8	2/1	Not fully consistent (2/3)
YAN (mg/L)	269.8 ± 16.1	276.2 ± 23.1	6.4 ± 15.8	2/1	Not fully consistent (2/3)
Aroma precursors					
3MH-Cys (μg/L)	7.3 ± 1.8	55.0 ± 9.3	47.7 ± 7.9	3/0	Consistent (3/3)—increase
3MH-Glu (μg/L)	75.0 ± 26.5	135.6 ± 59.2	60.6 ± 41.4	3/0	Consistent (3/3)—increase

DP = direct pressing; PFM = pre-fermentation maceration; ^1^ expressed in tartaric acid; SD = standard deviation. Given that the experimental design relied on biological replicates across vintages (n = 3), consistency in the sign of the difference across vintages is reported as an indicator of trend robustness rather than as a formal statistical test.

**Table 2:** Composition of Divona wines and effect of maceration (DP and PFM), AF temperature (15 °C and 20 °C) and the maceration × AF temperature interaction.

	Overall Mean ± SD	Maceration		AF Temperature		Maceration × AF Temperature	
		*p*-Value	Signif.	*p*-Value	Signif.	*p*-Value	Signif.
General composition							
Alcohol [%vol]	14.1 ± 0.3	0.733	ns	0.027	*	0.879	ns
Dry extract [g/L]	18.2 ± 0.9	0.782	ns	0.839	ns	0.879	ns
pH	3.55 ± 0.11	0.723	ns	0.027	*	0.573	ns
Total acidity ^1^ [g/L]	4.4 ± 0.4	0.723	ns	0.405	ns	0.573	ns
Acetic acid [g/L]	0.5 ± 0.1	0.555	ns	0.221	ns	0.573	ns
Tartaric acid [g/L]	1.6 ± 0.7	0.813	ns	0.329	ns	0.573	ns
Malic acid [g/L]	1.3 ± 0.8	0.782	ns	0.985	ns	0.879	ns
Glycerol [g/L]	7.6 ± 0.4	0.978	ns	0.405	ns	0.679	ns
CIELab coordinates							
L* [-]	99.1 ± 0.9	0.723	ns	0.405	ns	0.573	ns
a* [-]	−1.3 ± 0.3	0.723	ns	0.405	ns	0.939	ns
b* [-]	7.2 ± 2.0	0.723	ns	0.594	ns	0.573	ns
C* [-]	7.3 ± 2.1	0.723	ns	0.594	ns	0.573	ns
H* [-]	100.2 ± 0.7	0.733	ns	0.594	ns	0.573	ns
Aromatic compounds							
3MH [ng/L]	246.9 ± 223.3	0.733	ns	0.594	ns	0.879	ns
cis-Linalool oxide [µg/L]	8.6 ± 6.4	0.961	ns	0.594	ns	0.603	ns
trans-Linalool oxide [µg/L]	11.1 ± 8.5	0.813	ns	0.613	ns	0.879	ns
Linalool [µg/L]	35.8 ± 25.8	0.733	ns	0.822	ns	0.879	ns
α-Terpineol [µg/L]	17.9 ± 6.6	0.782	ns	0.594	ns	0.879	ns
β-Citronellol [µg/L]	17.5 ± 2.9	0.723	ns	0.594	ns	0.939	ns
Nerol [µg/L]	24.1 ± 20.1	0.994	ns	0.594	ns	0.573	ns
Geraniol [µg/L]	81.1 ± 48.9	0.782	ns	0.594	ns	0.879	ns

SD = standard deviation; DP = direct pressing; PFM = pre-fermentation maceration; ^1^ expressed as tartaric acid; Signif. codes: ‘*’ 0.05 ; ns = not significant.

**Table 3:** Sensory analysis of Divona wine–effect of maceration, AF temperature and the maceration × AF temperature interaction on the sensory profile of Divona wines.

	Overall Mean ± SD	LMM Retained Model	Maceration		AF Temperature		Maceration × AF Temperature	
			*p*-Value	Signif.	*p*-Value	Signif.	*p*-Value	Signif.
Color intensity	4.6 ± 0.5	without TankID	0.912	ns	0.973	ns	0.437	ns
Bouquet								
Fruity	4.4 ± 0.6	Full	0.977	ns	0.973	ns	0.243	ns
Floral	3.0 ± 0.9	Full	0.912	ns	0.973	ns	0.243	ns
Vegetal	1.9 ± 0.8	without TankID	0.977	ns	0.973	ns	0.262	ns
Mineral	1.5 ± 0.6	without TankID	0.912	ns	0.973	ns	0.871	ns
Stress	1.5 ± 0.7	without TankID	0.912	ns	0.973	ns	0.125	ns
Buttery/Milky	1.2 ± 0.4	without TankID	0.912	ns	0.973	ns	0.623	ns
Empyreumatic	1.2 ± 0.5	without TankID	0.992	ns	0.973	ns	0.774	ns
Bouquet quality	4.5 ± 0.5	Full	0.977	ns	0.973	ns	0.262	ns
Mouthfeel								
Acidity	4.8 ± 0.5	Full	0.912	ns	0.973	ns	0.601	ns
Volume	4.1 ± 0.4	Full	0.912	ns	0.973	ns	0.703	ns
Equilibrium	4.4 ± 0.5	Full	0.977	ns	0.973	ns	0.262	ns
Bitterness	2.4 ± 0.8	without TankID	0.912	ns	0.973	ns	0.157	ns
Overall appreciation	4.4 ± 0.5	Full	0.912	ns	0.973	ns	0.243	ns

SD = standard deviation; LMM = linear mixed model; TankID = maceration × AF temperature × vintage; ns = not significant.

**Figure A1:**
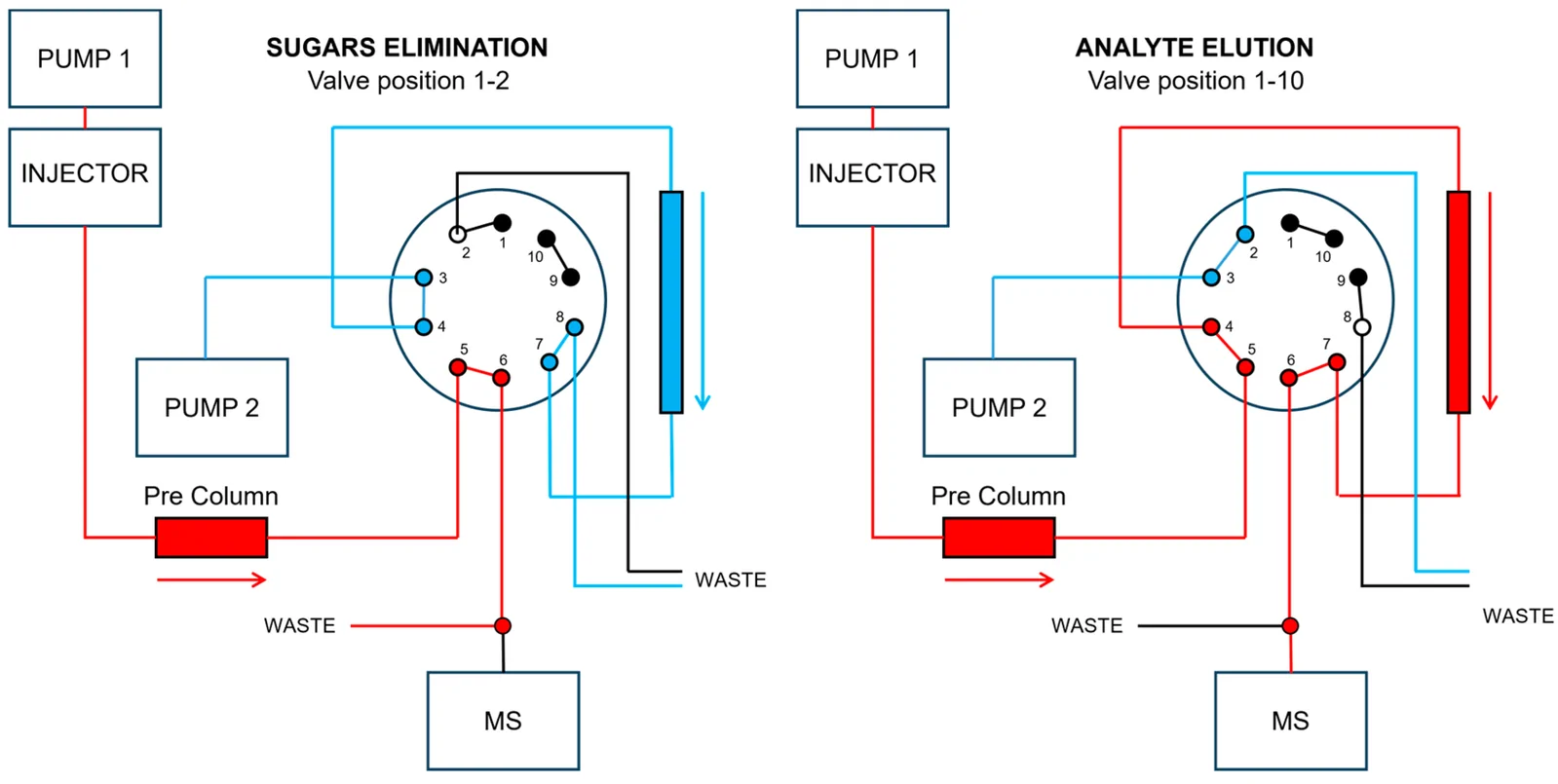
Connection schema of the column selection valve in the column compartment of the Agilent Infinity 1290 UPLC system, used for the quantification of 3MH-Cys and 3MH-Glu in must. Connections to the quaternary pump (Pump 1) are shown in red, while connections to the isocratic pump (Pump 2) are shown in blue. The schema illustrates the process starting with sugar elimination via the pre-column, followed by the elution of analytes from the analytical column.

## Data Availability

The original contributions presented in the study are included in the article, further inquiries can be directed to the corresponding author.

## Abbreviations

The following abbreviations are used in this manuscript:

3MH	3-mercaptohexanol
ANOVA	ANalysis Of VAriance
AF	Alcoholic fermentation
BH	Benjamini–Hochberg
DP	Direct Pressing
DTDP	4,4-dithiopyridine
ESI+	Electrospray Positive ionization
FTIR	Fourier Transform Infrared Spectroscopy
IST	Internal Standard
LC-MS	Liquid Chromatography–Mass Spectrometry
MFA	Multiple Factor Analysis
MRM	Multiple Reaction Monitoring
N_AA_	Amino acid nitrogen
PFM	Pre-Fermentation Maceration
SD	Standard Deviation
UPLC	Ultra-Performance Liquid Chromatography
YAN	Yeast Assimilable Nitrogen

## Appendix B

**Table A1:** Estimates of the fixed effects in the non-linear mixed-effects model for the parameters of the sugar curve.

Parameter	Effect	Estimate	Std Error	DF	t Value	*p*-Value
A	Intercept	112.53	1.49	141	75.73	<0.001
B	Intercept	−8.56	0.54	141	−15.73	<0.001
x_mid_	Intercept	3.99	0.14	141	28.08	<0.001
	AF Temperature (15 °C)	0.57	0.13	141	4.22	<0.001
	Maceration (PFM)	0.05	0.13	141	0.35	0.724
	Interaction	−0.04	0.13	141	−0.28	0.778
τ	Intercept	1.59	0.06	141	25.48	<0.001
	AF Temperature (15 °C)	0.36	0.05	141	7.41	<0.001
	Maceration (PFM)	−0.03	0.05	141	−0.68	0.496
	Interaction	−0.06	0.05	141	−1.24	0.216

**Table A2:** Results of pairwise comparisons (AF temperature) on a day-by-day basis, adjusted with the Benjamini–Hochberg procedure.

Days After Yeasting	Adjusted *p*-Value	Significance
0	1.000	ns
1	0.689	ns
2	0.689	ns
3	0.025	*
4	0.004	**
5	0.002	**
6	0.002	**
7	0.002	**
8	0.002	**
9	0.004	**
10	0.012	*
11	0.170	ns
12	0.768	ns

Signif. Codes: ‘**’ 0.01 ‘*’ 0.05, ns = not significant.
